# Changes in mouse whole saliva soluble proteome induced by tannin-enriched diet

**DOI:** 10.1186/1477-5956-8-65

**Published:** 2010-12-15

**Authors:** Elsa Lamy, Gonçalo Graça, Gonçalo da Costa, Catarina Franco, Fernando Capela e Silva, Elvira Sales Baptista, Ana Varela Coelho

**Affiliations:** 1 ESHTE - Escola Superior de Hotelaria e Turismo do Estoril, Estoril, Portugal; 2 ICAAM-Instituto de Ciências Agrárias e Ambientais Mediterrânicas, Universidade de Évora, Évora, Portugal; 3 ITQB-Instituto de Tecnologia Química e Biológica, Oeiras, Portugal; 4 Departamento de Biologia, Universidade de Évora, Évora, Portugal; 5 Departamento de Zootecnia, Universidade de Évora, Évora, Portugal; 6 Departamernto de Química, Universidade de Évora, Évora, Portugal

## Abstract

**Background:**

Previous studies suggested that dietary tannin ingestion may induce changes in mouse salivary proteins in addition to the primarily studied proline-rich proteins (PRPs). The aim of the present study was to determine the protein expression changes induced by condensed tannin intake on the fraction of mouse whole salivary proteins that are unable to form insoluble tannin-protein complexes. Two-dimensional polyacrylamide gel electrophoresis protein separation was used, followed by protein identification by mass spectrometry.

**Results:**

Fifty-seven protein spots were excised from control group gels, and 21 different proteins were identified. With tannin consumption, the expression levels of one α-amylase isoform and one unidentified protein increased, whereas acidic mammalian chitinase and Muc10 decreased. Additionally, two basic spots that stained pink with Coomassie Brilliant Blue R-250 were newly observed, suggesting that some induced PRPs may remain uncomplexed or form soluble complexes with tannins.

**Conclusion:**

This proteomic analysis provides evidence that other salivary proteins, in addition to tannin-precipitating proteins, are affected by tannin ingestion. Changes in the expression levels of the acidic mammalian chitinase precursor and in one of the 14 salivary α-amylase isoforms underscores the need to further investigate their role in tannin ingestion.

## Background

Saliva is an important fluid that rapidly adjusts to changes in dietary conditions. Salivary glands are mainly under nervous system control, and the composition of salivary secretions is rapidly altered over a wide range in response to various stimuli. Saliva serves as a physiological buffer against variations between the external and internal milieus. Such variations may be reflected in different salivary protein profiles resulting from different dietary habits. It was proposed that saliva protein composition varies also considerably among species, reflecting diverse diets and modes of digestion [1]. Animals using identical feeding niches may present similarities in their salivary protein composition, whereas the presence of particular proteins may be specific for particular feeding niches. For example, salivary amylase levels correlate with starch levels in each animal species' diet [2]. Moreover, salivary protein composition is modulated by diet. One example is the induction of salivary cystatins in rats ingesting capsaicin-containing diets [3]. Studies in humans have demonstrated changes in the salivary proteome induced by different basic tastes [4].

Several other studies on the adaptation of salivary protein composition to diet have investigated tannins (for review, see [5]). Tannins are plant secondary metabolites (PSMs) found in most food and drinks of vegetable origin, with a high capacity to bind proteins, polysaccharides, carbohydrates, and other macromolecules. Particularly with proteins, tannins may form stable complexes that tend to precipitate [6]. Tannins highly influence diet selection, and their presence may result in food avoidance attributable to either their astringent properties or detrimental post-ingestive effects [7].

Feeding tannins to mice and rats induces a considerable amount of a particular group of salivary proteins--the proline-rich proteins (PRPs) [8,9]--that protect animals against the negative post-ingestive effects of tannin and appear to reduce the aversive bitter or astringent properties of tannins [8-10].

Although PRPs, and particularly those belonging to the basic subgroup, appear to be the most effective tannin-binding salivary proteins [6] and the first line of defense to tannin ingestion, other less abundant salivary proteins may also be affected by tannin consumption. For example, histatins constitute a group of relatively small proteins with high tannin affinity. However, their presence was only found in saliva from humans and some primates [11]. The presence of tannin-binding salivary proteins other than PRPs has also been suggested in some herbivores, although these proteins have not been characterized [12].

Our group has been developing research on the relationship between salivary protein composition and dietary choice. In our previous one-dimensional electrophoresis study [13], we observed an increase in the expression level of one α-amylase isoform, suggesting that the effects of tannin ingestion on mouse salivary protein composition go beyond the increase in salivary PRPs. Our hypothesis is that expression of other salivary proteins would also change as a consequence of tannin ingestion. The present study sought to augment the knowledge of such putative changes in the salivary protein profile by using two-dimensional electrophoresis (2-DE) coupled with mass spectrometry. Such an approach has already been used in studies of human and small ruminant salivary proteomes, resulting in a high number of proteins identified and a more reliable protein expression comparison [14-17].

Although mouse has developed into a premier mammalian model system for exploring potential causes and treatments for human disease when human experimentation is not feasible or ethical, to our knowledge an overall characterization of this species' saliva proteome had not yet been made. Mouse saliva and salivary glands have been studied for diet-induced changes, and 2-DE maps of rodent saliva have been reported for rat parotid [18] and submandibular [19] saliva. However, no extensive protein characterization was performed in any of these studies. Mice have been used traditionally in studies concerning the complex physiological ingestive and digestive systems, and such protein characterization is important.

We used 2-DE coupled with mass spectrometry (matrix-assisted laser desorption ionization time-of-flight mass spectrometry [MALDI TOF MS] and MALDI TOF-TOF MS/MS) to characterize the mouse soluble fraction of whole saliva (SFWS) and to study the effects of tannin ingestion. The changes induced by tannin-enriched diets were assessed after tannin-protein insoluble complexes were removed. Histology of salivary gland morphology was performed to confirm the effects of tannin levels used in the experiments.

## Materials and methods

### Animals

Twelve inbred male Balb/c mice, five weeks of age, were obtained from the licensed bioterium of Instituto Gulbenkian de Ciência (Oeiras, Portugal). The animals were housed in type IV mouse cages (Techniplast; six mice per cage), according to European Union recommendations and the revision of Appendix A of the European Convention for the Protection of Vertebrate Animals used for Experimental and Other Scientific Purposes (ETS No. 123). Animals were maintained on a 12 h/12 h light/dark cycle at a constant temperature of 22°C with *ad libitum *access to water and a standard diet with 21.86% crude protein (dry basis) in the form of pellets (RM3A-P; Dietex International, Essex, UK). The animals were subjected to a 7 day acclimation period to minimize the effects of stress associated with transportation, followed by a 7 day pretrial period to allow adaptation to the ground diet used during the feeding trials. The standard pellet diet was ground daily with a blender [13]. Before the feeding trial period, the animals were individually weighed and allocated to two experimental groups, with no significant differences in body mass (25.5 ± 1.7 g). All animal procedures were approved by the scientific committee, were supervised by a scientist trained by the Federation of European Laboratory Animal Science, and conformed with Portuguese law (Portaria 1005/92), which followed European Union Laboratory Animal Experimentation Regulations.

### Feeding Trials

A 10 day experimental period was initiated immediately after the pretrial period. The control group (*n *= 6) received a tannin-free diet consisting of the same standard ground diet administered during the pretrial period. The quebracho group (*n *= 6) received the standard ground diet enriched with quebracho. Quebracho (Tupafin-Ato, SilvaChimica SRL, Cuneo, Italy) is a natural extract obtained directly from quebracho wood and is sold commercially, mainly for use in the leather industry. These extracts are commonly used in herbivore feeding studies as a model of condensed tannins (e.g., [20]). According to the manufacturer's information, the extract contains 72 ± 1.5% condensed tannins with a small amount of simple phenolics. This product was added to the standard diet to obtain a mixture that contained 7 g tannin/100 g wet weight, which is a dosage previously found to induce PRPs in mouse salivary glands [9]. The diets were prepared daily, and food and water were provided *ad libitum*.

### Saliva and salivary gland collection and sample preparation

After the 10 day feeding trial (day 11) individual mouse whole saliva secretion was induced with an intraperitoneal injection of pilocarpine and collected by aspiration from the mouth as described elsewhere [13]. Prior to protein quantification, saliva samples were centrifuged at 16,000 × *g *for 5 min at 4°C to remove particulate matter and salivary proteins that could be precipitated because they form a complex with tannins. Only the soluble fraction was used for further analyses. After saliva collection, the animals were euthanized with an overdose of xylazine hydrochloride combined with ketamine hydrochloride. The parotid glands were dissected, washed briefly with 0.1 M phosphate buffer, pH 7.4, and fixed in 10% neutral buffered formalin for routine histology.

### Histology

To confirm that the quebracho doses used in this study did, in fact, affect the salivary glands, parotid morphology was observed by light microscopy using a Nikon Eclipse 600 microscope (Kanagawa, Japan). After embedding the fixed parotid glands in paraffin wax using routine procedures, a series of 5 μm thick sections were cut with a microtome, and the slides were stained with hematoxylin and eosin. For each animal, 10 digital pictures from random areas of the parotid glands were collected with a Nikon DN 100 camera (Kanagawa, Japan) at 200× magnification. For each animal, the areas and perimeters of a minimum of 100 acini were randomly chosen and measured using SigmaScan Pro 5.0 software (SPSS, Chicago, IL, USA).

### Separation by two-dimensional gel electrophoresis (2-DE)

The soluble fraction of whole saliva (SFWS) protein concentration was determined using the bicinchoninic acid method (Pierce, Rockford, IL, USA), with bovine serum albumin (BSA) as the standard.

Individual mouse SFWS samples (*n *= 6) containing 100 μg total protein were mixed with rehydration buffer [17]. Samples were subjected to isoelectric focusing (IEF: first dimension) at 20°C in 13 cm IPG strips, pH 3-10, NL (Amersham Biosciences Europe GmbH, Freiburg, Germany) using an IPGphor Isoelectric Focusing System (Amersham Biosciences Europe GmbH, Freiburg, Germany). The following IEF program was used: 2 h at 0 V, 12 h at 30 V (active rehydration), 1 h at 200 V, 1 h at 500 V, 1 h at 1000 V, 1 h at a 1000-8000 V linear gradient, and 6 h at 8000 V. After focusing, proteins in the IPG strips were equilibrated and horizontally applied on top of a 12% sodium dodecyl sulfate polyacrylamide gel electrophoresis (SDS-PAGE: second dimension) gel (1 × 160 × 200 mm) [17]. Broad range molecular mass markers (Ref 161-0317; BioRad, CA, USA) were run simultaneously with the samples to calibrate the molecular masses of protein spots. Gels were stained with Coomassie Coloidal G-250 [21]. Additionally, a PRP-specific stain/destain procedure [22] was used in some gels to assess the induction of these proteins by tannins.

### Gel analysis

Digital 2-DE gel images were acquired using a scanning densitometer with internal calibration (Molecular Dynamics, Amersham Biosciences Europe GmbH, Freiburg, Germany) with LabScan software (Amersham Biosciences Europe GmbH, Freiburg, Germany). Gel analysis was performed using Image Master Platinum v.6 software (Amersham Biosciences Europe GmbH, Freiburg, Germany). Spot volume normalization in the various 2-DE maps was performed using relative spot volumes (% vol). Spot detection was first performed in automatic mode, followed by manual editing for spot splitting and noise removal. The gel containing the greatest number of protein spots for each diet condition was chosen as the reference gel. All other gels from the same experimental condition were matched to the reference gel by placing user landmarks on approximately 10% of the visualized protein spots to assist in automatic matching. After completion of automatic matching, all matches were checked for errors by manual editing.

### Protein identification

#### In-gel digestion

Stained spots were excised, washed in acetonitrile, and dried in a SpeedVac. The proteins were digested with trypsin as previously described [23].

#### Peptide mass fingerprinting

Peptide mass fingerprinting was performed as described elsewhere [23], with mass spectra obtained by matrix-assisted laser desorption/ionization-time of flight mass spectrometry (MALDI TOF MS) using a Voyager-DE STR (Applied Biosystems, Foster City, CA, USA) MALDI TOF mass spectrometer in the positive ion reflectron mode. Database searches were performed against SwissProt, MSDB, and NCBInr following the same criteria described previously [23], both to perform the search and to accept the identification.

#### Protein identification using MALDI TOF-TOF data

Protein identification was performed by MALDI TOF-TOF analysis using an Applied Biosystems 4800 Proteomics Analyzer (Applied Biosystems, Foster City, CA, USA) in both MS and MS/MS mode. Positively charged ions were analyzed in the reflectron mode over the *m/z *range of 800-3500 Da, typically using 800 laser shots per spectra and a fixed laser intensity of 3500 V. External calibration was performed using the 4700 Calibration Mix (Applied Biosystems). The 10 best s/n precursors from each MS spectrum were selected for MS/MS analysis by Collision-Induced Dissociation assisted with air using a collision energy of 1 kV and a gas pressure of 1 × 10^6 ^torr. Two thousand laser shots were collected for each MS/MS spectrum using a fixed laser intensity of 4500 V. Raw data were generated by 4000 Series Explorer v3.0 RC1 software (Applied Biosystems, Foster City, CA, USA). All contaminant *m/z *peaks were included in the exclusion list used to generate the peptide mass list for the database search. The generated mass spectra were used to search UniProtKB (released July 7, 2009) and Uniref100 (released July 7, 2009). Searches were conducted using two algorithms: Paragon from Protein Pilot v.2.0 software (Applied Biosystems, MDS Sciex) and Mowse from MASCOT-demon v.2.1.0 software (Matrix-Science, London, UK). Protein identifications were accepted with a probability filter cutoff of 99% (Prot Score ≥ 2.0) for Paragon and 95% (*p *< 0.05) for Mowse. For Protein Pilot, the search parameters were the following: enzyme (trypsin), Cys alkylation (iodoacetamide), special factor (urea denaturation), species (none), and ID focus (biological modification). For Mascot, the interpretation of the combined MS+MS/MS data was performed using GPS Explorer v.3.5 software (Applied Biosystems, Foster City, CA, USA), with the following parameters: missed-cleavage (one), peptide tolerance (75 ppm), fragment mass tolerance (0.25 Da), fixed modification (carbamidomethylation of cysteine), and variable modification (methionine oxidation). Additionally, all MS/MS spectra were further analyzed with Peaks Studio v.4.5 software (Bioinformatics Solutions, Waterloo, ON, Canada) for automatic *de novo *sequencing combined with database searching, selecting trypsin as the enzyme and a parent and fragment mass error tolerance of 0.08 U.

### Prediction of post-translational modifications

For salivary α-amylase, which was identified in several spots for which apparent molecular masses and *pI *differed, potential post-translational modifications (PTMs) were predicted as described previously [17]. Briefly, FindMod (http://www.expasy.ch/tools/findmod/; accessed June 17, 2010), NetPhos 2.0 (http://www.cbs.dtu.dk/services/NetPhos/; accessed June 17, 2010), and Signal IP 3.0 (http://www.cbs.dtu.dk/services/SignalP/; accessed June 17, 2010) search engines were used. Glycosylation and phosphorylation information present in the SwissProt database were also considered. Only the predicted PTMs associated with peptides not matched to the identified protein were considered.

### Statistical analysis

All data were analyzed for normality using the Kolmogorov-Smirnoff test and homoscedasticity using the Levene test. The values of salivary protein concentration were normally distributed, and independent sample *t*-tests were performed to assess differences between diet treatments. Spot relative volume (% vol) and parotid acinar areas and perimeters did not present normal distributions or homoscedasticity. Consequently, the differences in the expression levels between the control and quebracho groups for each protein spot and for histomorphometric data were determined using the nonparametric Mann-Whitney test. Means were considered significantly different when *p *< 0.05. All statistical analyses were performed using SPSS v.15.0 software (SPSS, Chicago, IL, USA).

## Results

### Histology

**Figure 1:**
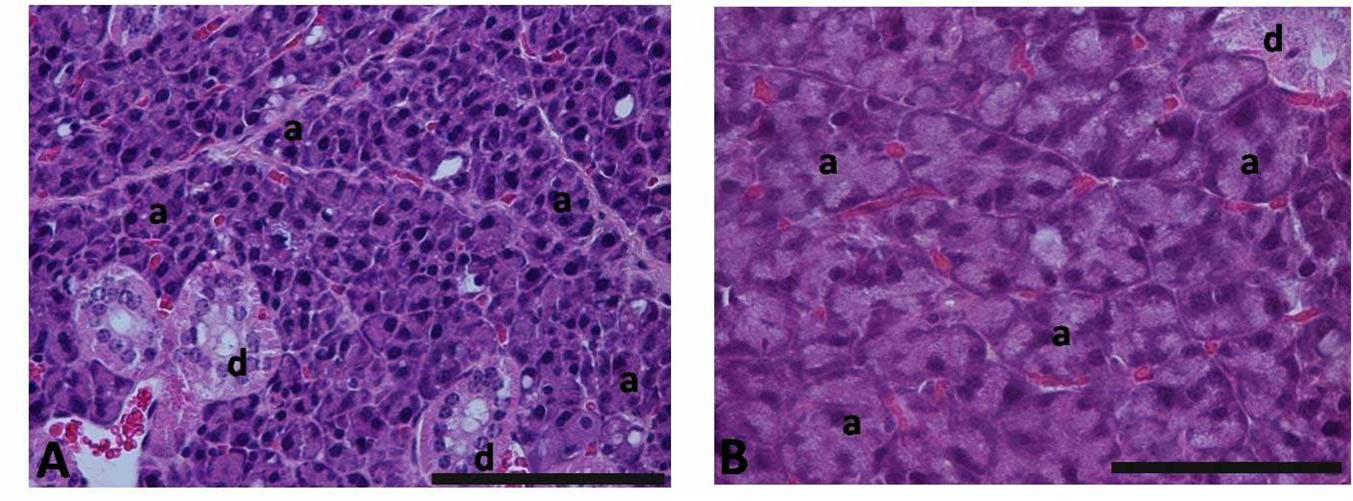
**Examination by ligth microscopy of parotid glands**. Original magnification X200 (bar = 50 μm). Acini from control group (A) are significantly lower than those from quebracho tannin-enriched diet group (B). a - acinus; d - salivary ducts.

The acinar area and perimeter of the parotid glands from the animals fed a quebracho-enriched diet were significantly higher than the control group (Table 1). Levels of 7 g tannin per 100 g wet weight in the diet produced hypertrophy of parotid gland secretory tissue (Figure 1).

**Table 1:** Comparison of parotid histomorphological parameters and saliva protein concentration between mice control (n = 6) and quebracho groups (n = 6)

Parameters	Control Group	Quebracho Group	Significance
**Area **(Pixel)	13,415 ± 4,334	33,353 ± 14,413	13.0^a^

**Perimeter **(Pixel)	464 ± 78	739 ± 160	12.9^a^

**Protein concentration **(μg/mL)	2920 ± 289	1941 ± 138	0.00012^b^

Values presented correspond to mean ± standard deviation

***^a ^***Differences are significant for Z > 1.96

^b ^Differences are significant for P < 0.05

### Pattern of soluble fraction of whole saliva (SFWS) proteins

After the 10 day feeding trial, the protein concentration of the soluble fraction of whole saliva, measured after centrifugation and precipitate removal, was significantly lower in the quebracho group than in the control group (Table 1), indicating that a smaller amount of salivary proteins remains soluble after tannin ingestion.

**Figure 2:**
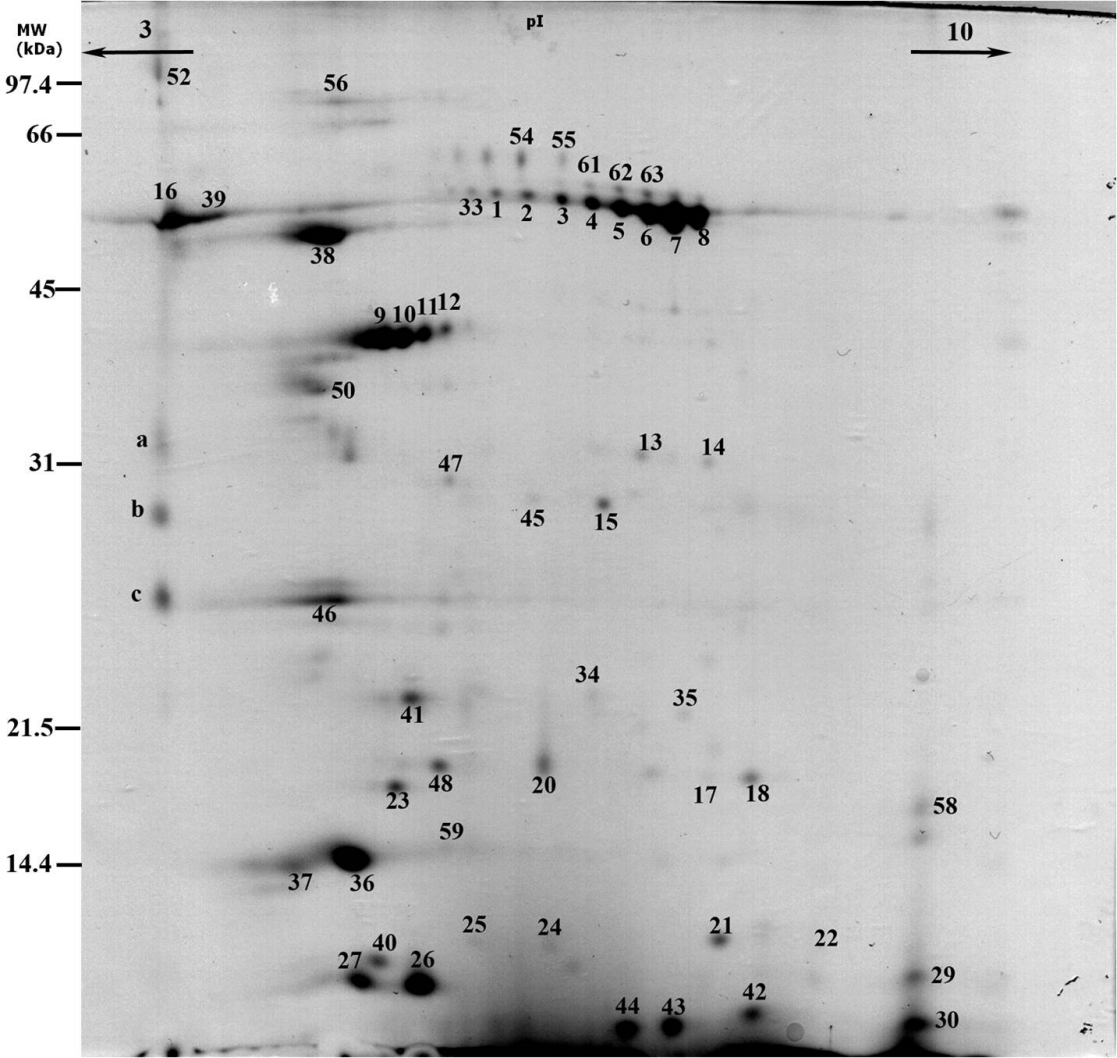
**Two-dimensional proteome profile of mice whole saliva**. Aliquots containing 100 μg of proteins from control animals were subjected to IEF in a 3-10 NL range, separated by molecular masses in 12% polyacrylamide gels and stained with Coomassie Coloidal G-250. Molecular markers masses are represented on the left side of the gel. Numbered protein spots were collected for protein identification.

A two-dimensional map of mouse SFWS was constructed with a non-linear *pI *range of 3-10 and a molecular mass ranging from approximately 10 to 100 kDa (Figure 2). A total of 86 protein spots were reproducibly displayed in Coomassie Colloidal G-250-stained gels, from which the 57 most intense ones were analyzed by mass spectrometry. From these, 48 protein spots, corresponding to 21 polypeptides, were identified by peptide mass fingerprinting and MS/MS (Table 2). Some proteins were identified in a high number of spots, namely salivary amylase (14 different spots), androgen binding proteins (6 different spots), and several forms of kallikreins (8 different spots). Concerning salivary amylase, glycosylation and deamidation are predicted PTMs, according to the analysis of the mass spectra and presence of consensus regions (Table 3).

**Table 2:** Proteins from the soluble fraction of mice whole saliva identified by Mass spectrometry

Protein name	Swiss-Prot accession number	Spot	Est. MW (kDa)/pI	Theor. MW (kDa)/pI	Identified by	
					**PMF Score**^a^	**MS/MS Score**^b^
Acidic mammalian chitinase	CHIA_MOUSE	38	53/4.8	50/4.9	170	694
				
		56	97/5.0		---	192

Androgen binding protein alpha	Q9WUM8	36	15/5.0	10/5.4	78	---

Androgen binding protein beta	Q8R1E9	26	11/5.2	12/5.1	102	394
				
		27	11/5.0		80	140

Androgen binding protein gamma	Q8JZX1	42	11/7.2	13/7.7	121	---
				
		43	10/6.1		157	546
				
		44	10/6.0		69*	442

Carbonic anhydrase VI	CAH6_MOUSE	9	44/5.0	35/5.8	177	428
				
		10	44/5.1		156	637
				
		11	44/5.2		193	699
				
		12	44/5.3		134	535

Cysteine-rich secretory protein 1	CRIS1_MOUSE	13	33/6.0	26/4.5	82*	243
				
		14	33/6.3		79*	107

Dcpp1 protein	Q64097	34	21/5.9	18/6.1	70*	---
					
		35	20/6.2		82*	

Dcpp2 protein	Q6PCW3	18	17/6.8	16/7.8	97	421

Deoxyribonuclease-1	DNAS1_MOUSE	50	39/4.9	30/4.7	90	300

Glandular Kallikrein K1	KLK1_MOUSE	20	18/5.7	26/4.9	79	476
				
		41	21/5.1		---	113
					
		a	33/3.4			110

Glandular Kallikrein K13	P36368	40	12/5.1	26/8.3	71	---

Glandular Kallikrein K5	K1KB5_MOUSE	47	32/5.4	26/5.3	109	514

Glandular Kallikrein K9	K1KB9_MOUSE	21	12/6.4	26/7.2	84	283

Glandular Kallikrein K22	K1B22_MOUSE	15	30/5.9	26/6.0	111	300
				
		45	31/5.6		77*	---

Muc 10	Q8VC95	30	11/9.2	20/10.2	85*	---

Odorant binding protein Ia	P97336	23	17/5.2	17/5.2	182	761

Odorant binding protein Ib	P97337	48	18/5.4	17/5.5	118	506

Parotid secretory protein	PSP_MOUSE	46	25/4.9	23/5.0	82	776
				
		c	25/3.4		---	360

Prolactin-inducible protein homolog	PIP_MOUSE	37	15/4.8	14/4.8	---	107

Salivary amylase	AMY1_MOUSE	1	58/5,5	56/6.5	88	887
				
		2	58/5,6		193	834
				
		3	58/5,7		193	777
				
		4	58/5,8		217	772
				
		5	58/5,9		230	738
				
		6	58/6,0		270	795
				
		7	58/6,1		226	632
				
		8	58/6,2		232	804
				
		16	58/3,4		155	632
				
		33	58/5,4		107	774
				
		39	58/3,7		148	825
				
		61	58/5,8		132	927
				
		62	58/5,9		163	853
				
		63	58/6,0		149	795

Vomeromodulin precursor	Q80XI7	54	66/5.6	61/5.5	81#	---
					
		55	66/5.7		68#	

^a ^Score is significant (p < 0.05) when higher than 75 (search performed with no taxonomic restriction), 67 (* search restricted to mammal database), or 61 (# search restricted to rodent database);

^b ^Total Ion Score is a result of the sum of all ion scores of the fragmented ions. Ions were only considered for protein identification if a significant ion score was obtained (p < 0,05).

**Table 3:** Alpha-amylase predicted posttranslational modifications

Spot	PTM	
	
	N-glyc.^1^	Deamidation^2^
1	----	417; 419; 431
		
2	427; 475	-----
		
3	----	417; 419; 431
	
4	475	364; 365; 379
	
5	427; 475	364; 365; 379; 417; 419; 431
	
6	----	364; 365; 379
		
7	475	364; 365; 379
		
39	427; 475	----

Here residues are indicated for which PTMs are predicted, according with mass spectra analysis

^1 ^Based on the existence of a consensus region and the absence of the peptide from tryptic peptide maps

^2 ^Based on FindMod and the absence of the peptide from the tryptic peptide maps

### Effects of quebracho consumption on SFWS protein patterns

**Figure 3:**
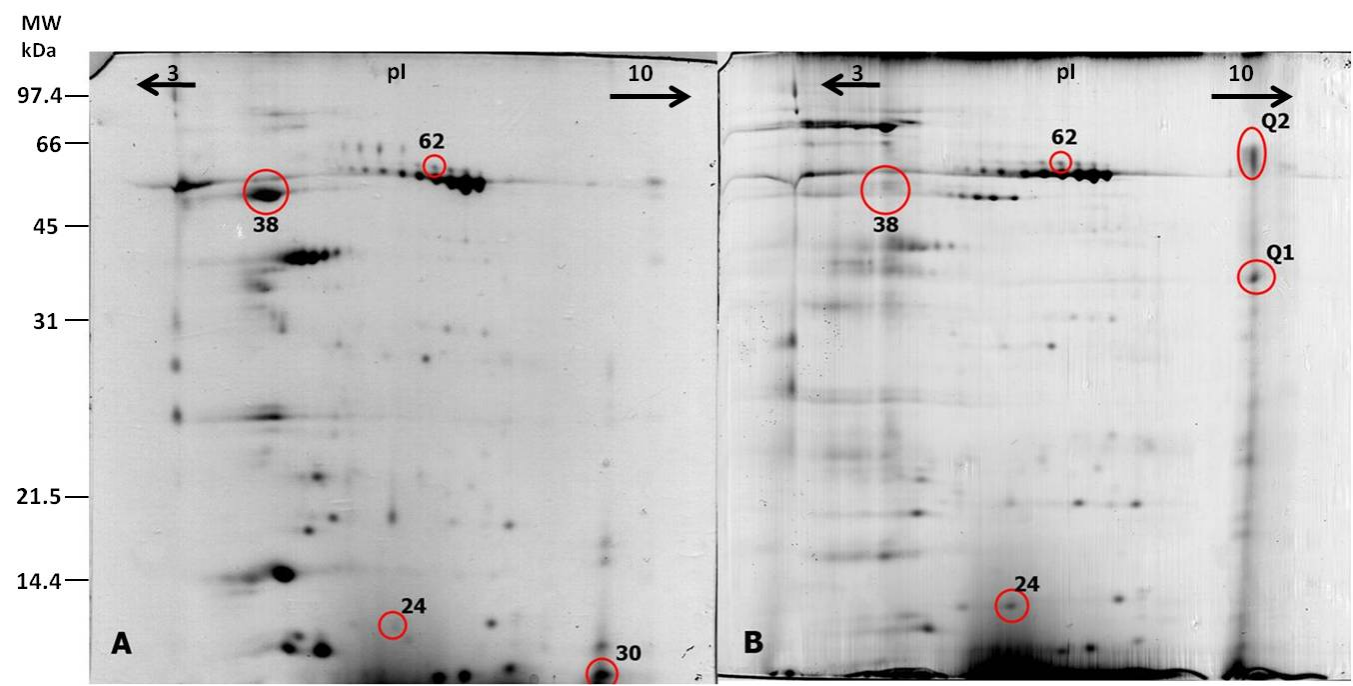
**Changes in the proteome of mice whole saliva after quebracho consumption**. A) representative gel of an individual from control group; B) representative gel of an individual of quebracho group. A decrease in relative volume was observed for spot 38, whereas spots 24 and 62 increased after quebracho consumption. Spot 30 was only observed in the gels from control group, whereas spots Q1 and Q2 were only observed in 2-DE gels from quebracho group. These last spots were dark pink stained. Coloured pictures are presented in supplementary Figure S1. Some of the spots for which expression levels are different in this figure did not change consistently in all individuals and, consequently, are not discussed.

Three protein spots were found to change significantly in terms of relative volume (Table 4). The levels of one isoform of α-amylase (spot 62) and one unidentified protein (spot 24) increased in the quebracho group, whereas the levels of acidic mammalian chitinase (spot 38) decreased. Two new protein spots (Q1 and Q2), which were not observed in the control group gels, were consistently present in the gels from the quebracho group, whereas spot 30, corresponding to Muc 10, was absent (Figure 3). For other protein spots, no statistical significant differences were determined due to high variability between individuals in their expression levels.

**Table 4:** Comparison of the expression levels of selected proteins (%Vol, mean ± SD) between control (N = 6) and quebracho-fed animals (N = 6)

Spot N°	Control group	Quebracho group	P*^a^*	Protein
**24**	17.07 ± 41.23	110.87 ± 59.55	0.0099	Not identified

**38**	475.26 ± 272.43	110.99 ± 132.25	0.015	Acidic mammalian chitinase precursor

**62**	0.28 ± 0.02	0.44 ± 0.05	0.00015	Salivary amylase

***^a ^***Differences are significant for P < 0.05

When both the control and quebracho gels were subjected to the Coomassie Brilliant Blue R-250 modified staining procedure for PRPs [22], the Q1 and Q2 spots, present at the basic extremity of the gels and with apparent molecular masses of 42 and 64 kDa, respectively, appeared with a slightly dark pink color, whereas the remaining spots appeared as blue spots in both gels (Additional file 1: supplementary Figure S1).

## Discussion

Using 2-DE and MS/MS, a proteome profile of mouse SFWS comprising 21 different proteins was established, extending knowledge and aiding studies on ingestive physiology and salivary secretion physiology using a mouse model.

Williams et al. [18] obtained 2-DE maps from rat parotid saliva using a *pI *range similar to that used in the present study (pH 3-10). Comparisons with our gels indicate a similar distribution for some spots, notably those we identified as α-amylase, deoxyribonuclease, parotid secretory protein, and demilune cell and parotid protein. However, several differences observed between the two patterns are not surprising because they stem from distinct genotypes (rat *vs*. mice) and different glandular origin secretions (parotid *vs*. whole saliva). The presence of basic and acidic PRPs was suggested in a rat parotid saliva 2-DE pattern [18]. These proteins are not constitutively expressed in mouse salivary glands [9], but rather induced by isoproterenol administration or tannin ingestion [5,9], which can also explain the failure to detect them in our control samples.

In the present study, different spots resulted in the identification of the same protein (Figure 2; Table 2). A similar feature was also observed in the human saliva proteome [24] and recently by us in small ruminant parotid saliva [17]. This observation may be attributable to the presence of isoforms, protein fragments, or PTMs, among which glycosylation and phosphorylation were reported to be a common feature of salivary proteins [25,26].

Fourteen spots were identified as salivary α-amylase. A high number of salivary α-amylase spots with a similar distribution has also been observed in human whole saliva [15,16,27], suggesting similarities between humans and mice in the digestive functions of saliva, in contrast to ruminants, which lack salivary α-amylase [17,22]. The apparent molecular masses were approximately 58 kDa, and the *pI *ranged from 3.4 to 6.2. The theoretical molecular mass of the native form of α-amylase is 56 kDa, with a *pI *of 6.4. Glycosylation with neutral and acidic (sialic acid) oligosaccharides and spontaneous post-secretion deamidation of salivary α-amylase have been previously demonstrated [27,28]. The consensus sequence for glycosylated asparagine residues is Asn-X-Ser/Thr; therefore, two possible *N*-glycosylation sites for mouse α-amylase are 427-429 and 475-477 (Table 3). These two sites were also previously mentioned for human [27,28] salivary α-amylase. The described glycosylations may explain the higher apparent molecular mass of the α-amylase isoforms compared with the native α-amylase form and the *pI *differences between the several protein spots. Moreover, from the MS spectra analysis of the several spots identified as α-amylase, the absence of one or both peptides containing the consensus region for *N*-glycosylation was apparent, suggesting the possibility of such a PTM. No potential phosphorylations were observed for the protein spots identified as α-amylase, and the presence of the signal peptide was predicted for all of the spots.

Eight protein spots, from acidic to neutral and with several different molecular masses, were identified as five different kallikrein forms. These proteins belong to a family of serine proteases that are involved in hormone and growth factor processing [29]. The tremendous amount and diversity of kallikreins present in mouse saliva was not previously observed using SDS-PAGE [13]. Some authors already reported the expression of several kallikreins in mouse submandibular glands [30]. Besides, these salivary proteins were also found in humans [31] and rats [32]. The relative proportion of the various tissue kallikreins secreted by rat submandibular glands was found to be differentially influenced by the two branches of the autonomic nervous system: kallikreins in sympathetic-induced saliva were derived by exocytosis of pre-packaged granules in granular tubules, whereas kallikreins in parasympathetic-induced saliva were likely secreted through a constitutive vesicular route [33]. In the present study, we used pilocarpine to stimulate mouse saliva secretion, and we hypothesize that the identified forms derive mainly from a constitutive vesicular route. This may be important for future studies that apply parasympathetic agonists to stimulate salivary flow and collect saliva.

When tannins were introduced in the diet, changes in mouse SFWS 2-DE profiles were observed. Tannins are generally believed to be synthesized by plants to act as deterrents because of their bitter and astringent properties [34,35]. The challenge is to ingest plant-derived foods without suffering the ill effects of tannins. Saliva components are the first defense line against tannins, partially by minimizing their unpalatable astringent properties [10]. Hypertrophy of parotid glands was reported in rats and mice, which coincided with a dramatic increase in salivary PRP production after 2-3 days of tannin ingestion [8,9]. Similar results were found in the present study, where a significant increase in acinar size was observed in animals fed quebracho tannin-enriched diets.

The protein concentration of the SFWS from the quebracho group was lower compared with the control group, suggesting that the stable insoluble complexes formed between salivary proteins and dietary tannins in the mouth [36] were lost during the centrifugation step during sample preparation, which was intentional and allowed us to analyze the minor expressed salivary proteins.

In the present work, only one mucin was identified in mouse SFWS. Mucins represent a high proportion of salivary proteins (approximately 16% of the total proteins in human whole saliva), play a protective role, and contribute to oral coating and lubrication [37]. The failure to identify mucins may be related to the difficulty of assessing these proteins because of their large molecular mass, high viscosity, and poor solubility in aqueous solvents [38]. The Muc10 spot, although present in the control group, was absent in the 2-DE profile of mouse SFWS from the quebracho group. Salivary mucins can also form complexes with tannins [39], and Muc10-tannin complexes may have been removed during the centrifugation step.

The level of chitinase decreased in the quebracho group. The presence of this protein in mouse saliva was already observed by one-dimensional electrophoresis, but its level did not change after tannin consumption [13]. This protein has been reported in mice [40] and humans [41,42], and a digestive or defensive role against chitinous pathogens [40-42] has been proposed. Future studies may clarify the biological role of this protein in tannin ingestion.

From the protein spots that newly appeared in the quebracho group, spots Q1 and Q2 stained dark pink (Additional file 1: supplementary Figure S1), suggesting that these may be PRPs [21]. The proteins present in these two spots were not identified by mass spectrometry. A failure in identifying PRPs by mass spectrometry was previously reported [43], which the authors attributed to the particular characteristics of this family of proteins. Identification of PRPs by mass spectrometry is challenging because of the primary sequence of these proteins, for which tryptic digests produce only a reduced number of high *m/z *values [43]. This behavior also causes difficulties when extracting the peptides from the gel. In fact, the peptide maps for both spots are similar and poor with regard to the number of *m/z *peaks detected. The location of the Q1 and Q2 spots at the basic extremity of the gels is consistent with studies arguing that basic PRPs act as a defense mechanism against dietary tannins [6,36]. Moreover, their induction was observed in salivary glands of polyphenol-fed rats [8] and BALB/c mice [9]. Salivary basic PRPs have a very high affinity for tannins, leading to the formation of insoluble complexes [6]. The observation of pink spots even after centrifugation also demonstrates the presence of free PRPs or PRP-tannin soluble complexes [44].

The increase in expression level of one α-amylase isoform was previously observed in mice fed tannin-enriched diets [13]. This increase was suggested to be a co-adjuvant of the inhibition of tannin biological activity or as a response to counteract the amount of this enzyme that was potentially inactivated by tannin binding [13]. Inhibition of salivary α-amylase by dietary polyphenols has been demonstrated [45], and a recent report revealed the mechanisms involved in the tannin/α-amylase interaction [41]. The observation of changes in only one of the isoforms, previously [13] and in the present study, supports the hypothesis of different functional activities among the several isoforms. Further studies are needed to elucidate the functional differences between amylase isoforms.

## Conclusion

The present study characterized the mouse saliva protein profile, which is an animal model used in studies of salivary gland physiology. Salivary protein composition correlates with systemic conditions, and the knowledge of its normal composition may elucidate the differences induced by treatments. Despite the similarities to the extensively studied human saliva protein profile, significant differences were also found, demonstrating the species specificity of saliva. Additionally, we demonstrated that mouse SFWS 2-DE profile changes in response to introducing quebracho tannins into diet, namely by increasing the expression of one salivary amylase isoform and decreasing the expression of the acidic mammalian chitinase precursor. Because these are proteins which did not precipitate tannins, they may act through an alternative mechanism to impede them to have negative effects in the digestive tract. These findings suggest that salivary proteins other than PRPs may play a role in the modulation of saliva composition according to the characteristics of ingested material. Proteomics will be useful in nutrition studies for monitoring changes in saliva composition induced by foods with particular characteristics.

## List of abbreviations

PRP: Proline-rich protein; PSM: Plant Secondary Metabolites; 2-DE: Two-dimensional electrophoresis; MS: mass spectrometry; MS/MS: tandem mass spectrometry; MALDI TOF: Matrix assisted laser desorption ionization time-of-flight; BSA: bovine serum albumin; IEF: isoelectric focusing; IPG: Immobilized pH gradient; H&E: Hematoxylin and Eosin; SDS-PAGE: Sodium dodecyl sulphate polyacrylamide gel electrophoresis; CBB: Comassie Brilliant Blue; PTM: Post-translational modifications; SFWS: Soluble fraction of whole saliva

## Competing interests

The authors declare that they have no competing interests.

## Authors' contributions

All authors contributed and approved the final manuscript. ESB and AVC were responsible for the conception and design of the study. GG and GC performed the protein separation and protein identification by PMF. CF performed the MALDI TOF-TOF protein identification. Histologic studies were performed by EL and FCS. EL and GG analyzed and interpreted the data. EL drafted the manuscript and ESB and AVC revised it critically for important content. All authors confirm that the content has not been published elsewhere and does not duplicate their published work.

## Supplementary Material

Additional file 1 **Supplementary Figure S1 - Changes in the proteome of mice whole saliva after quebracho consumption**. Spots Q1 and Q2, which were only observed in 2-DE gels from quebracho group, appear dark pink following Beeley et al.^24 ^CBB R-250 stainning protocol for PRPs. Click here for file
