# Supplementary figures and images for: Changes in mouse whole saliva soluble proteome induced by tannin-enriched diet
Source: Proteome Sci. 2010 Dec 15;8:65. doi: 10.1186/1477-5956-8-65 (PMC3018447; doi:10.1186/1477-5956-8-65)

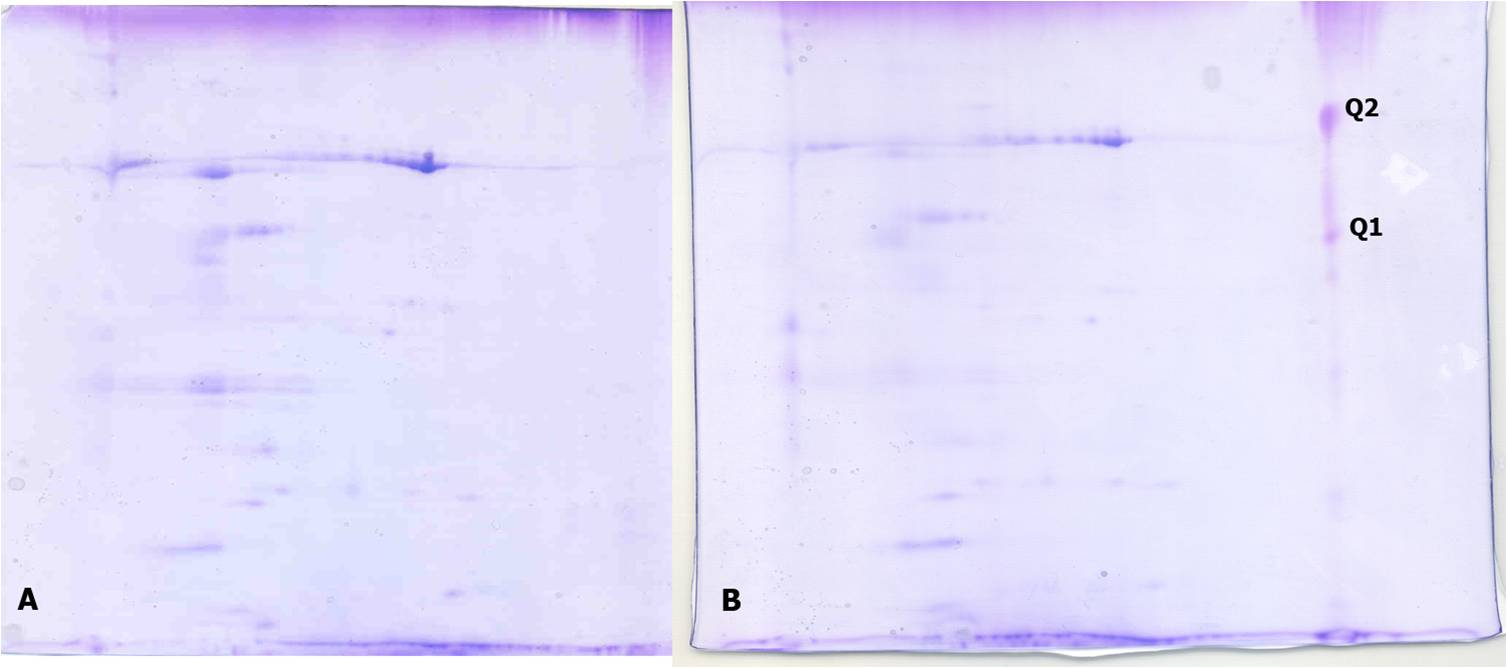

Supplement: Additional file 1 — Supplementary Figure S1 - Changes in the proteome of mice whole saliva after quebracho consumption. Spots Q1 and Q2, which were only observed in 2-DE gels from quebracho group, appear dark pink following Beeley et al.24 CBB R-250 stainning protocol for PRPs. [file 1477-5956-8-65-S1.JPEG]
